# An examination of sleep spindle metrics in the Sleep Heart Health Study: superiority of automated spindle detection over total sigma power in assessing age-related spindle decline

**DOI:** 10.1186/s12883-023-03376-3

**Published:** 2023-10-06

**Authors:** Kalyan Palepu, Kolia Sadeghi, Dave F. Kleinschmidt, Jacob Donoghue, Seth Chapman, Alexander R. Arslan, M. Brandon Westover, Sydney S. Cash, Jay Pathmanathan

**Affiliations:** 1 Beacon Biosignals, 22 Boston Wharf Rd 7th Floor, Suite 41, Boston, MA 02210 USA; 2 Beth Israel Deaconess Medical Center, 330 Brookline Ave, Boston, MA 02215 USA; 3 Clinical Data Animation Center (CDAC), Massachusetts General Hospital, 50 Staniford Street, Fruit St, Boston, MA 02114 USA

**Keywords:** Sleep Spindles, Sigma Power, Sigma Coherence, EEG, Spindle coherence, Drug development

## Abstract

**Background:**

Sleep spindle activity is commonly estimated by measuring sigma power during stage 2 non-rapid eye movement (NREM2) sleep. However, spindles account for little of the total NREM2 interval and therefore sigma power over the entire interval may be misleading. This study compares derived spindle measures from direct automated spindle detection with those from gross power spectral analyses for the purposes of clinical trial design.

**Methods:**

We estimated spindle activity in a set of 8,440 overnight electroencephalogram (EEG) recordings from 5,793 patients from the Sleep Heart Health Study using both sigma power and direct automated spindle detection. Performance of the two methods was evaluated by determining the sample size required to detect decline in age-related spindle coherence with each method in a simulated clinical trial.

**Results:**

In a simulated clinical trial, sigma power required a sample size of 115 to achieve 95% power to identify age-related changes in sigma coherence, while automated spindle detection required a sample size of only 60.

**Conclusions:**

Measurements of spindle activity utilizing automated spindle detection outperformed conventional sigma power analysis by a wide margin, suggesting that many studies would benefit from incorporation of automated spindle detection. These results further suggest that some previous studies which have failed to detect changes in sigma power or coherence may have failed simply because they were underpowered.

## Statement of significance

Sleep spindles are transient oscillatory rhythms in the “sigma” band (10-16 Hz) distinct from the background non-rapid eye movement (NREM) EEG. They are preserved across mammalian sleep [1], but their physiological role is uncertain (roles in learning and memory consolidation, and sleep maintenance have been postulated). Changes in sleep spindle characteristics can be seen in a variety of disease processes, in normal aging, and with drug therapy. Sleep spindle activity is often assessed using total sigma band power in NREM2 sleep [2–12]. However, the distinctive morphological appearance of these transient events allows for automated segmentation using quantitative methods [13–15]. We show that sigma power over the entirety of NREM2 is a coarse and underpowered measurement. As such, we hypothesized that null results will be more common in experiments which aim to detect changes in spindle activity using this measurement, compared to measures using automated spindle isolation. We find that automated spindle detection is superior for feature characterization and allows detection of statistically significant sleep differences with smaller sample sizes. These results demonstrate the value of automated spindle detection for clinical trials and drug development.

## Introduction

Sleep spindles are bursts of rhythmic 10–16 Hz activity lasting 0.5–2 s that are the hallmark of stage 2 or “non-Rapid Eye Movement stage 2” (NREM2) sleep [16]. Spindles are thought to play a role in memory consolidation [17–21] and appear to be altered in a wide variety of neuropsychiatric conditions including autism [22], schizophrenia [23–25] and neurodegenerative diseases [26–28]. Given the importance of these graphoelements, much work has been invested in developing tools for spindle detection and characterization [29].

Power spectral density in the sigma range (10–16 Hz) computed over the entirety of NREM2 sleep (referred to here as “*NREM2 sigma*” power) and sigma band coherence (“*NREM2 sigma*” coherence) are common metrics used to quantify spindle presence and activity in sleep EEG (see review in Fernandez and Lüthi [5]), particularly in drug pharmacodynamic studies [2–4, 6–12, 30].

Nevertheless, NREM2 sigma power and coherence are coarse measures due to sparsity of spindle activity (and therefore sigma power) in the overall NREM2 or NREM timeframe. Spindles comprise a small portion of sleep, with NREM2 typically having fewer than two spindles per minute [31] (some studies estimate slightly higher rates; see [32, 33]), and fewer in NREM3 sleep. Additionally, sigma power is not exclusively driven by spindle activity, so sigma power computed over NREM2 sleep can be confounded by other EEG activity [16]. As a result, approaches averaging sigma power spectral features over large portions of sleep may wash out contributions from spindles, resulting in low statistical power and null results that are difficult to interpret. Consistent with this possibility, many studies using NREM2 sigma power have found no significant overall effects on sigma power, including studies of Suvorexant [7, 11, 34, 35] and several other sleep drugs [36, 37]. Some studies have aimed to resolve these problems by using automated spindle detection to focus only on portions of sleep containing spindles. For example, use of manual spindle counting found that zolpidem increased spindle count [21], and a similar result was found with eszopiclone using a wavelet automated detection algorithm [24]. Nevertheless, currently there is no standard approach to characterizing spindles in pharmacodynamic studies, and both approaches continue to be used.

We sought to investigate how much benefit an automated spindle detection algorithm adds over NREM2 sigma power and coherence measures, with a particular emphasis on hypothesis testing. To do this, we simulated clinical trials of varying sample sizes that attempted to discern the known age-related decline in spindle coherence, evaluating the necessary sample size using NREM2 sigma versus spindle sigma. We hypothesize a substantially smaller sample size requirement when using spindle sigma metrics calculated with automated methods.

## Methods

### Data

We used data from the Sleep Heart Health Study (SHHS) [38], a large, publicly available home PSG dataset, and included sleep EEG recordings from 5,793 patients over 40 years old [38, 39]. Electrode placement followed the 10–20 system, and all recordings included 2 electrodes placed at positions C3 and C4. The dataset has an additional 2,647 follow-up recordings which took place a minimum of 3 years after the patient’s first recording, leading to a total of 8,440 recordings. Sleep stages were manually scored in the original dataset by licensed sleep technicians using American Academy of Sleep Medicine standards [40]. Each epoch was staged as one of the following: wake (W), rapid eye movement, Non-REM stage 1 (N1), Non-REM stage 2 (NREM2 or NREM2), or Non-REM stage 3 (N3). These labels are publicly available.

### Spindle detection

**Fig. 1:**
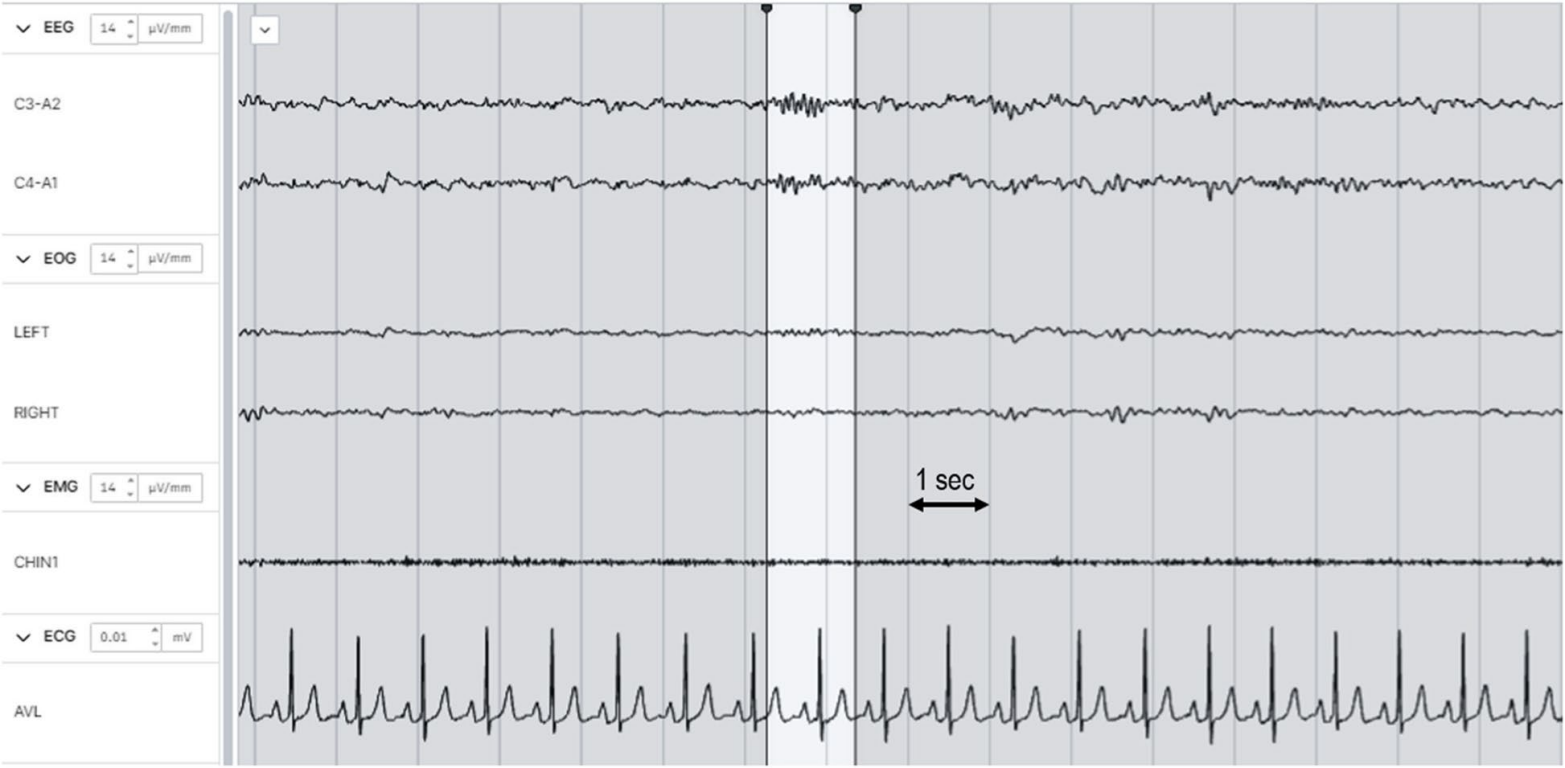
Example of an automated spindle detection. Vertical lines indicate 1 s intervals. Spindle segment is highlighted

The aforementioned data was ingested into the Beacon’s cloud platform, where automated spindle detection was performed using a previously published LUNA algorithm [31]. Signals were band-pass filtered between 0.3 and 35 Hz, artifacts were excluded using automated detection, and electrocardiogram (ECG) interference was subtracted prior to automated spindle detection, as per the LUNA algorithm. Spindle detection was limited to sleep segments staged as NREM2. All detection parameters were set to default values in LUNA, allowing for maximal generalizability as the default parameters have been optimized for general use (see: https://zzz.bwh.harvard.edu/luna/ref/spindles-so/, defaults listed in “Primary Options” table). Specifically, target spindle center frequency, fc = 13.5 Hz; cycles = 7; spindle “quality” metric based on the ratio of the relative sigma band power to the broadband power, q = 0; multiplicative threshold (core spindle amplitude exceeds “th” times the mean) = 4.5x; minimum spindle duration = 0.5 s; maximum spindle duration = 3 s; maximum seconds between spindle patterns, below which fragments should be merged, “merge” = 0.5 s. [31]. See Fig. 1 for a sample automated detection.

### Sigma power and coherence calculation

Each recording was split into 10 s epochs, and sigma power and coherence were computed for each epoch, where the sigma band was defined as power in the 10–16 Hz band. Sigma power was averaged across channels and calculated using the MNE-Python “psd_array_multitaper” function within the “time–frequency” suite. Coherence was calculated between EEG channels C3 and C4 using the MNE-Python “spectral_connectivity” within the “pactools” suite, multitaper mode [41]. Complex coherence was calculated using MNE-Python (see https://mne.tools/mne-connectivity/stable/generated/mne_connectivity.spectral_connectivity_epochs.html) as:

$$Coherence=\frac{abs(E\left[{S}_{C3C4}\right])}{\surd (E\left[{S}_{C3C3}\right]*E\left[{S}_{C4C4}\right])}$$

where E[S_C3C4_] is the cross spectral density of C3 and C4 channels averaged over all epochs, E[S_C3C3_] is the power spectral density of C3 averaged over all epochs, and E[S_C4C4_] is the power spectral density of C4 averaged over all epochs. Note that the recording specific sampling rate was used for each calculation (SHHS recordings varied from 125 to 250 Hz sampling rates, with the majority recorded at 125 Hz). The average per-patient *NREM2 sigma* power and coherence was compared to average per-patient *spindle sigma* power and coherence using an asymptotic two-sample KS-test.

### Statistical analysis

Statistical tests described above were performed using the HypothesisTesting.jl package in Julia. We set the significance threshold at the standard 5% level and report explicit *p*-values.

### Simulated clinical trials

To compare statistical power between *NREM2 sigma* power-based metrics and more targeted *spindle sigma* detection-based metrics we examined the difference between coherence calculated with *NREM2 sigma* and *spindle sigma* coherence to detect expected age-related changes in spindle coherence. Sigma coherence was selected for its robust age-related change between baseline and follow-up recordings, both when restricting to segments of NREM2 containing spindles and when looking at the entirety of NREM2 (see Fig. 4). For sample sizes ranging from 10 to 150, we simulated 100,000 random datasets by subsampling the required number of patients from the set of patients with follow-up recordings and performed a paired sample *t*-test on the difference in mean NREM2 sigma coherence between the baseline and follow-up recordings. We then repeated this, comparing spindle coherence. The power for each measure was the proportion of repetitions with a significant difference.

## Results

### Average per-patient spindle power and coherence compared to average per-patient sigma power and coherence in NREM2 sleep

**Fig. 2:**
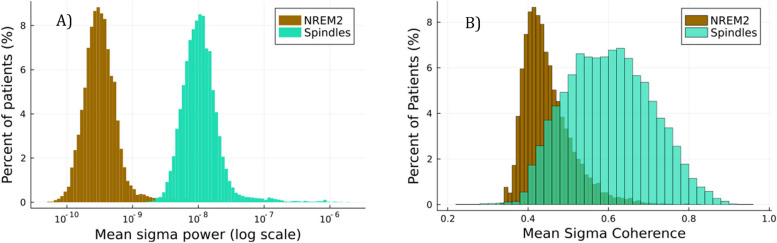
**A** Distribution of mean sigma power over either all of NREM2 sleep (N2 sigma), or restricted to automated spindle detections (spindle sigma). **B** Distribution of mean sigma coherence calculated by NREM2 sigma sleep or spindle sigma

We first examined spindle metrics using sigma power and coherence calculated across all NREM2 epochs (“*NREM2 sigma*”) compared to sigma power and coherence calculated across segments containing spindles (“*spindle sigma*”). Although spindles are seen in slow wave sleep as well, we focused on NREM2 given the higher spindle density and faster background frequencies (which would suggest the lowest difference between epoch level and spindle specific sigma band features), NREM2 spindles are better correlated with sleep macroarchitecture (such as NREM2, NREM3, and REM duration) whereas slow wave sleep spindles are not, and NREM2 spindles are more impacted by age [31]. Additionally, we did not distinguish between spindle subtypes, instead using the broad sigma band (10–16 Hz) to capture both fast and slow spindles. While we expected a substantial increase in spindle sigma power, the magnitude was unknown as was the result for coherence. Average per-patient *NREM2 sigma* power and coherence and *spindle sigma* power and coherence are shown in Fig. 2.

*NREM2 sigma* power had a mean of 7*.*4 × 10^−10^ μV^2^, while *spindle sigma* power was 1.4 × 10^–8^ μV^2^ – a 20-fold difference (Fig. 2A). Coherence was less differentiated, presumably due to relative left–right symmetry of sleep architecture at the central leads, albeit slightly higher when calculated with spindle sigma. Mean *NREM2 sigma* coherence was 0.44, while mean *spindle sigma* coherence was 0.58 (Fig. 2B). Both power and coherence distributions were significantly different (*p* < 0*.*001) for spindle versus NREM2 sigma.

### Age related decline in sigma coherence

**Fig. 3:**
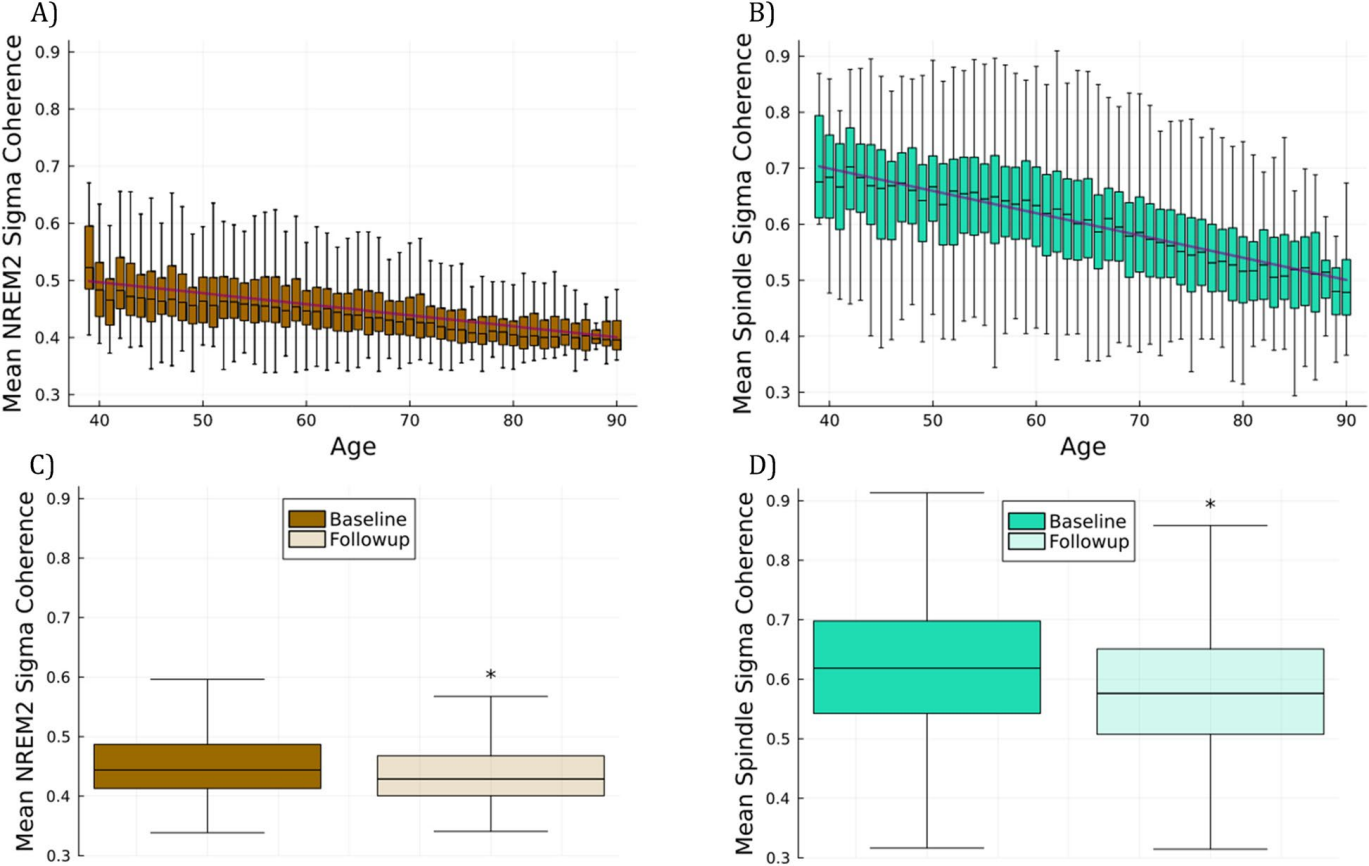
**A** Population NREM2 sigma coherence as a function of age. Note the drop in coherence over time. **B** Population spindle sigma coherence as a function of age shows a steeper slope. **C** Mean NREM2 sigma coherence at the time of the first test is significantly (*p* < 0.001) higher than at the time of repeat testing a minimum of 3 years later. **D** Same as **C** but using spindle sigma coherence

**Fig. 4:**
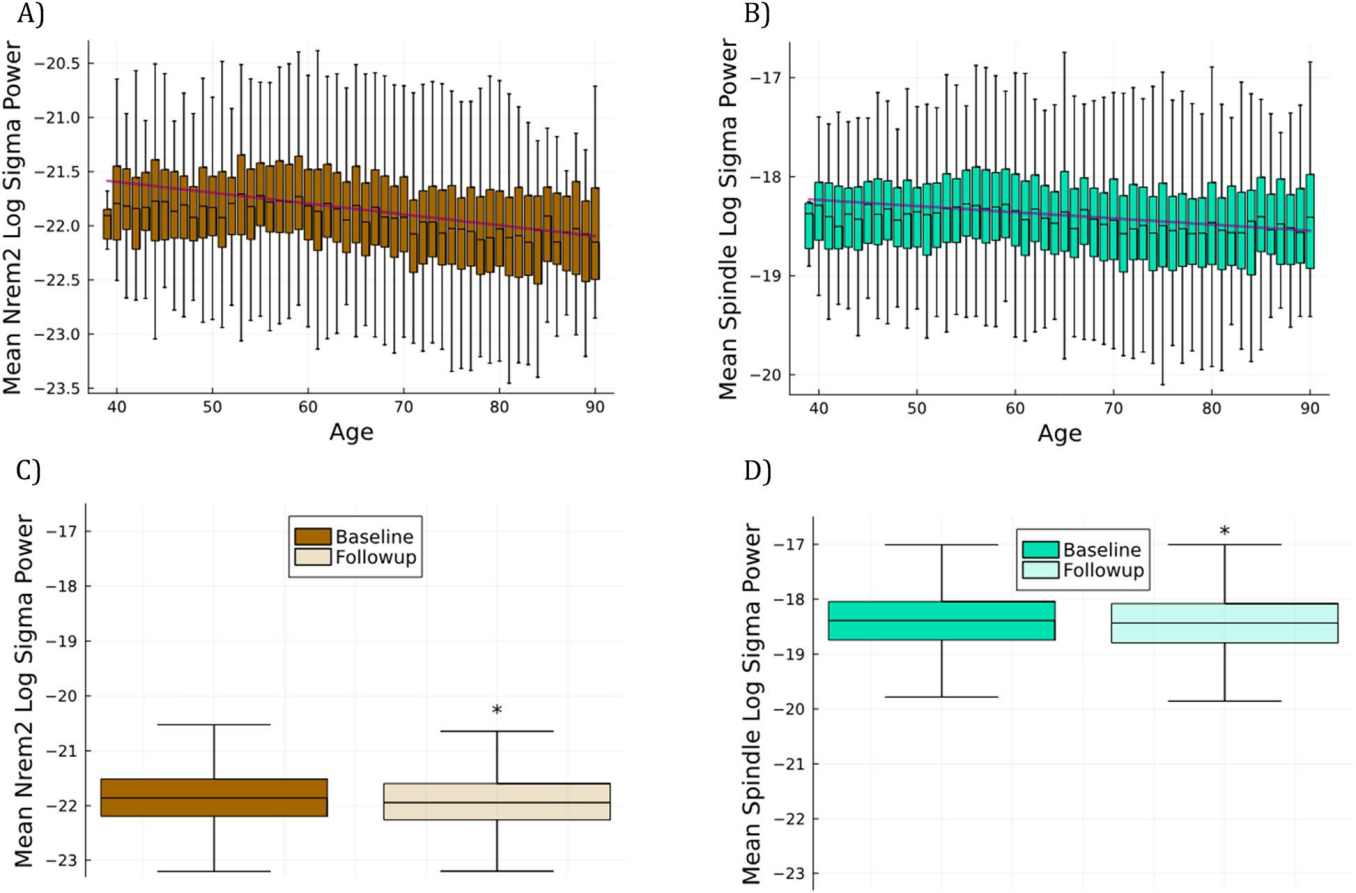
**A** Population NREM2 sigma power and **B** spindle sigma power as a function of age. **C** Mean N2 sigma power and **D** spindle sigma power are significantly reduced from baseline to follow up a minimum of 3 years later

Using the entire population of subjects with polysomnographic EEG data over two or more time points, we examined the change in *NREM2 sigma* and *spindle sigma* coherence with age. Spindles metrics are known to change with age, including power and coherence [31, 42], although age related effects on spindle coherence are not well described. In this dataset, at the population level both *NREM2 sigma* coherence and *spindle sigma* coherence showed a linear decline with increasing age at testing (Fig. 3A and B), although a steeper decline is found for spindle sigma coherence. Additionally, for the 2,647 individuals with repeat testing at a minimum of 3 years later, we found that mean coherence at baseline was 000 statistically significantly higher (*p* < 0.001) than the mean coherence at repeat testing for both *NREM2 sigma* and *spindle sigma* (0.02 for mean *NREM2 sigma*, and 0.04 for *spindle sigma*, Fig. 3C and D). A similar relationship was found for NREM2 sigma power and spindle sigma power (both showed a statistically significant decline at follow up a minimum of 3 years later), although the magnitude of age-related change was not as large as for coherence (Fig. 4).

### Simulated clinical trials

**Fig. 5:**
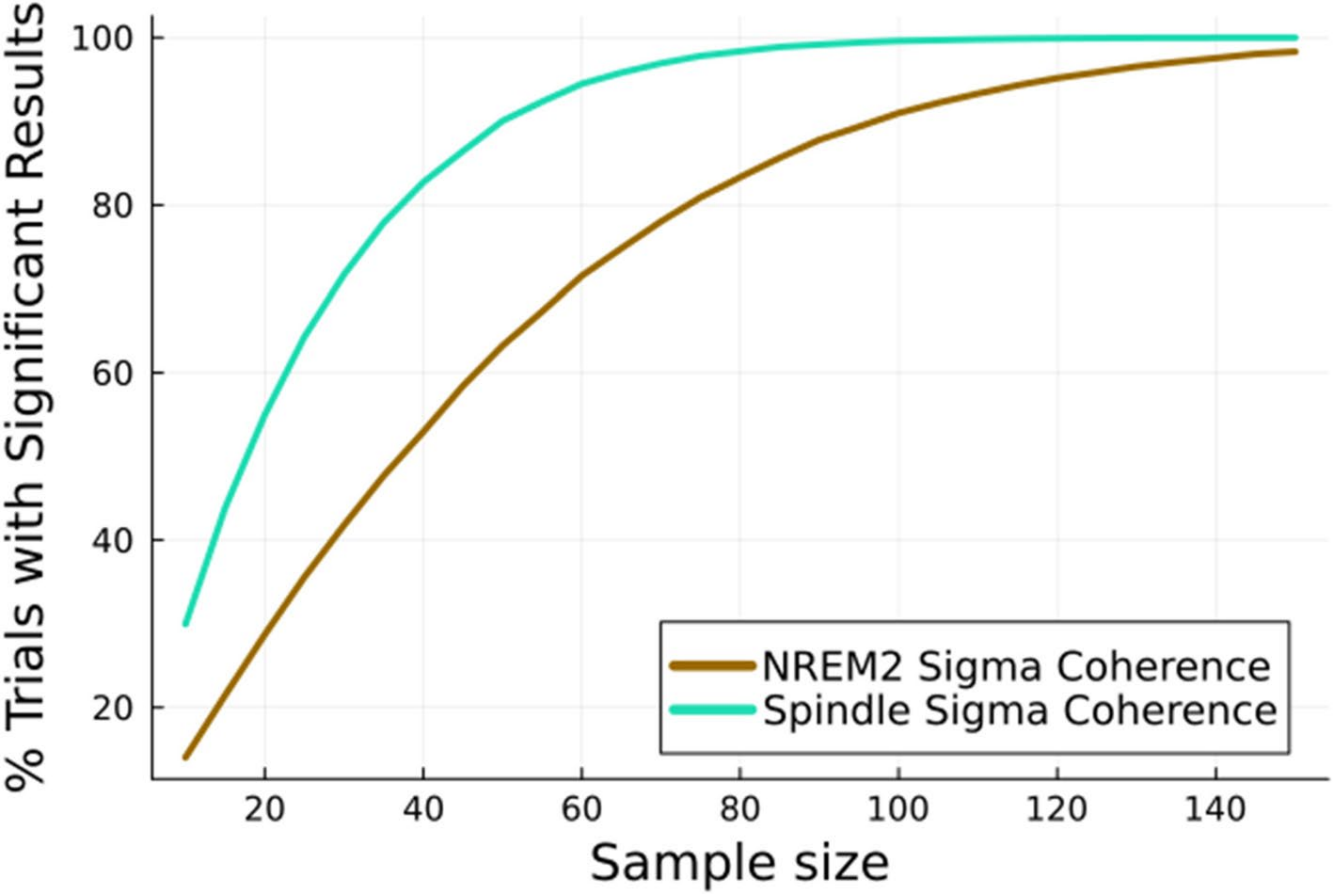
For each sample size, 100,000 clinical trials were simulated by randomly subsampling patients and performing a t-test to try to detect a change in sigma coherence between baseline and follow-up recordings. Calculating sigma coherence just for spindles instead of all NREM2 consistently resulted in a higher power

To evaluate the utility of *spindle sigma* over *NREM2 sigma* we simulated clinical trials attempting to demonstrate the age-related statistically significant decline in coherence demonstrated in Fig. 3. From the population of 2,647 subjects with repeat testing, we drew samples ranging from 10 to 150 subjects. For each sample size, the “clinical trial” was repeated 100,000 times and the percentage of trials that showed a statistically significant result was characterized (demonstrating the power of that sample size for the test measure). As shown in Fig. 5, most trials with sample sizes of less than 40 subjects failed to achieve statistically significant results when using *NREM2 sigma* coherence. In contrast, more than 80% of trials using *spindle sigma* coherence achieved significance. *Spindle sigma* consistently resulted in a higher percentage of simulated trials with significant results. Achieving 95% power required a sample size of 60 when relying on *spindle sigma* coherence, but nearly twice as many participants (115) when relying on *NREM2 sigma* coherence.

## Discussion

Our results show that sigma power and coherence over NREM2 miss critical information from sleep EEG. Mean sigma power and coherence calculated over all NREM2 sleep are much lower than they are over just the regions with detected spindles, demonstrating that averaging sigma power over all of NREM2 sleep washes out and thus conceals local peaks in sigma power and coherence. Given that *NREM2 sigma* power and coherence analyses struggle to distinguish sleep with and without spindles, it seems likely that these methods risk missing changes in analyses that aim to detect relatively minor changes in spindle density or morphology, such as pharmacodynamic studies done as part of drug development.

We note that some studies use relative sigma power (power in the 10–16 Hz band divided by power in the 0.3–30 Hz band) rather than absolute sigma power as in our experiments [32, 43]. We also note that our study used only one spindle detector, and results may vary depending on the detector or settings used. Nevertheless, this detector is readily available and the key issue is quite general: neglecting the distinction between periods that do vs. do not contain spindles reduces statistical power, and this applies whether using absolute or relative sigma power or reasonable spindle detectors with varying sensitivity or specificity.

Our results show that, at least in some cases, studies reporting statistically insignificant changes in NREM2 sigma power or coherence may be underpowered to detect true changes. For example, previous studies of suvorexant [7, 11, 35] or gaboxadol [3, 6] have used sigma power as a surrogate for spindle power, and found either no change or changes in specific cases only. Our results suggest that use of spindle power would allow for detection of a significant change that could be missed using global sigma power. In our data, a sample size of 70 was required for a beta power of 80% to detect age related spindle changes when using sigma coherence, which was almost double the size of the sample needed when using spindle coherence. The sample size necessary to achieve 95% beta power was twice as large for sigma-based methods as compared to targeted spindle-based methods. We suggest that these findings would translate to all studies seeking to assess changes induced changes in spindle characteristics.

## Data Availability

The datasets analyzed during the current study are available in the Sleep Heart Health Study repository, [https://sleepdata.org/datasets/shhs].
